# The problem of pseudoreplication in neuroscientific studies: is it affecting your analysis?

**DOI:** 10.1186/1471-2202-11-5

**Published:** 2010-01-14

**Authors:** Stanley E Lazic

**Affiliations:** 1 Cambridge Computational Biology Institute, Department of Applied Mathematics and Theoretical Physics, University of Cambridge, Cambridge, CB3 0WA, UK; 2 Oxford Centre for Collaborative Applied Mathematics, Mathematical Institute, University of Oxford, Oxford, OX1 3LB, UK; 3 Current address: F. Hoffmann-La Roche AG, In Silico Sciences - Statistics, 4070 Basel, Switzerland

## Abstract

**Background:**

Pseudoreplication occurs when observations are not statistically independent, but treated as if they are. This can occur when there are multiple observations on the same subjects, when samples are nested or hierarchically organised, or when measurements are correlated in time or space. Analysis of such data without taking these dependencies into account can lead to meaningless results, and examples can easily be found in the neuroscience literature.

**Results:**

A single issue of Nature Neuroscience provided a number of examples and is used as a case study to highlight how pseudoreplication arises in neuroscientific studies, why the analyses in these papers are incorrect, and appropriate analytical methods are provided. 12% of papers had pseudoreplication and a further 36% were suspected of having pseudoreplication, but it was not possible to determine for certain because insufficient information was provided.

**Conclusions:**

Pseudoreplication can undermine the conclusions of a statistical analysis, and it would be easier to detect if the sample size, degrees of freedom, the test statistic, and precise *p*-values are reported. This information should be a requirement for all publications.

## Background

**Figure 1:**
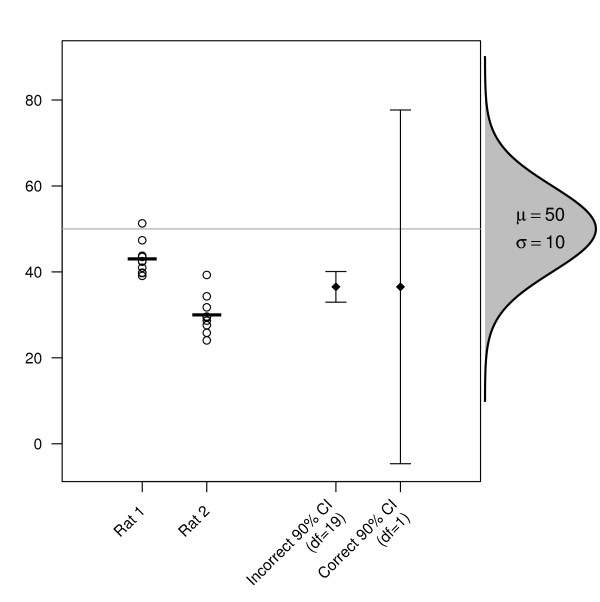
**An example of pseudoreplication**. Two rats are sampled from a population with a mean (*μ*) of 50 and a standard deviation (*σ*) of 10, and ten measurements of an arbitrary outcome variable are made on each rat. The first (incorrect) 90% CI uses all 20 data points and does not account for the hierarchical nature of the data. For the second 90% CI, the mean of the ten values for each rat are calculated first, and then only these two averaged values are used for the calculation of the CI. The error bar on the left is incorrect because each of the 20 data points are not a random sample from the whole population, but rather samples within two rats. This is evident from the fact that the 10 points are normally distributed around the mean of their respective rats, but not normally distributed around the population mean (horizontal grey line), as would be expected when independent samples are randomly drawn from a population. Increasing the number of observations on each rat does not lead to a more precise estimate of *μ*, which requires more rats. Note that 90% CI are plotted for clarity because the graph needs to be greatly compressed to display the 95% CI.

The majority of neuroscience experiments include some type of inferential statistical analysis, where conclusions are reached based on the distance between the observed results from some hypothetical expected value. Discovering how the brain and nervous system work requires the proper application of statistical methods, and inappropriate analyses can lead to incorrect inferences, which in turn leads to wasted resources, biases in the literature, fruitless explorations of non-existent phenomena, distraction from more important questions, and perhaps worst of all, ineffectual therapies that are advanced to clinical trials [1,2]. Pseudoreplication is a particularly serious error of analysis that has not received much attention in the neuroscience literature, and which Hurlbert defined over twenty years ago as the "... use of inferential statistics to test for treatment effects with data from experiments where either treatments are not replicated (though samples maybe) or replicates are not statistically independent" [3]. Put simply, it is a confusion between the number of data points with the number of independent samples, and can be illustrated with the following example. Suppose the following information was provided in the Methods section of a manuscript: "Ten rats were randomly assigned to either the treatment or the control group, and performance on the rotarod (a test of motor coordination) was tested on all the rats on three consecutive days. Differences between groups were assessed with a two-tailed independent samples *t*-test, with *p *< 0.05 considered statistically significant." Then in the Results section the authors report that "the treatment group did significantly better than the control group (*t*_28 _= 2.1; *p *= 0.045)." Have the authors analysed the data correctly? No. With a sample size of ten rats, there should only be eight degrees of freedom (*df *= *n*_1 _+ *n*_2 _- 2, where *n*_1 _and *n*_2 _are the number of independent samples in each group) associated with this statistical test. The concept of degrees of freedom is perhaps not the most intuitive statistical idea, but it can be thought of as the number of independent data points that can be used to estimate population parameters (e.g. means, differences between means, variances, slopes, intercepts, etc.), and whenever something is estimated from the data, a degree of freedom is lost. Therefore the total *df *is equal to the sample size only if all the samples are independent: measuring the height of ten unrelated individuals provides ten independent pieces of information about the average height of the population from which they were drawn; measuring the height of one person ten times provides only one independent piece of information about the population. In the above rat example, the three observations from each rat (from the three days of testing) were treated as independent samples, and hence the 28 degrees of freedom arose from thinking that the fifteen data points in each group contain independent information about the effect of the treatment (*n*_1 _= *n*_2 _= 15, and so 15 + 15 - 2 = 28). Incidentally, the correct analysis with a *t*-statistic of 2.1 on 8 *df *has a corresponding *p*-value of 0.069. In addition to the incorrect degrees of freedom, there is also the problem of false precision, which is discussed at greater length below (see Figure 1). However, it should be noted here that the *df *problem has greater relevance when sample sizes are small, but false precision is arguably of greater concern in general.

The assumption of independence means that observations within each group or treatment combination are independent of each other. An alternative way of expressing this concept is to say that the errors (residual values) are independent, once the effects of all the other explanatory variables have been taken into account. In addition, other variables that are not included in the analysis (e.g. the order in which the samples were obtained) must not influence the outcome or be correlated with the residuals. The remainder of the introduction will define some commonly used terms, illustrate why pseudoreplication is problematic, and finally, discuss the four situations in which it can arise.

The terms *sample*, *replicate*, *observation*, *experimental unit*, *n*, and *experiment *have overlapping meanings, are often used interchangeably, and can have different meanings based on the context. An *experimental unit *is defined as the smallest entity that can be randomly assigned to a different treatment condition [4]. A person or a rat are typical experimental units, because they can be allocated to different treatments. The *sample size *is usually reported as the "*n*" and is defined as the number of experimental units, but the term is slightly ambiguous because one could take two blood samples from a rat (in the morning and afternoon for example) and therefore there are twice as many samples as rats, but the "sample size" still refers to the number of rats. An *observation *occurs whenever a value of an outcome variable is recorded, and it is equivalent to the number of data points; if there are twenty rats and only one observation is taken on each rat, then the number of observations equals the sample size (*n*). If multiple observations are taken from each rat, then observations within each rat are not independent and therefore all of the observations cannot be summed to give a total sample size. In cell culture experiments, the whole procedure is often repeated three or more times and reported as three "independent replicate experiments". In this case *n *is the number of experiments. The term experiment is ambiguous in this context because all of the independent trials or runs taken together can be thought of as "the experiment". *Replicates *also typically refers to independent observations, and hence the term pseudoreplication when this is not the case. However, Cumming et al. use replicates to refer to "repetition of measurements on one individual in a single condition, or multiple measurements of the same or identical samples", and thus they use the term replicate to refer to observations that are not independent [5]. The difference here is between *biological replicates *which are independent (e.g. two unrelated rats are biological replicates) and *technical replicates *which are not independent (e.g. dividing a blood sample from a single rat into two sub-samples, and measuring the concentration of a substance in each sub-sample). In this paper, replicates refers to biological replicates unless otherwise indicated; references to samples or sample size (*n*) refer to the number of independent values, and observations are used to refer to individual data points, which most likely are not independent (but observations could be independent if there is only one observation per animal for example). The term pseudoreplication is used synonymously with lack of independence of observations, correlated observations, and correlated errors.

### Pseudoreplication leads to the wrong hypothesis being tested and false precision

Ignoring lack of independence leads to two major problems. The first is that the statistical analysis is not testing the research hypothesis that the scientist intends, in other words, the incorrect hypothesis is being tested. This is illustrated in Figure 1, where two rats are sampled from a population, and the interest is in determining whether the rats come from a population with a mean of 50 on some arbitrary outcome variable (shown horizontally on the right), or is their value far enough away from 50 that we conclude that they come from a different population. This can be stated as *H*_0_: *μ *= 50 (the null hypothesis), and *H*_0_: *μ *≠ 50 (alternative hypothesis). Ten measurements are made on each rat and a one-sample *t*-test can be used to compare the mean of this single sample of rats to a hypothesised population value. The incorrect analysis would give *t*_19 _= -7.75, a *p*-value of 2.7 × 10^-7^, and a 95% confidence interval (CI) from 32.9 to 40.2. The correct analysis would give *t*_1 _= -2.07, *p *= 0.287, and 95% *CI *= (-46.3, 119.4). The change in *p*-value between the two analyses is six orders of magnitude, which demonstrates the importance of dealing with pseudoreplication appropriately. When calculating standard errors and confidence intervals, and making inferences between different groups with statistical tests, the assumption is that all the values are independently drawn from the parent population, but clearly the rat that the observation came from partly determines what that value is. Statistical analyses performed on such data without regard for this structure are often meaningless (in this case the researcher would falsely conclude that the mean of the sample is less than 50). The incorrect 95% confidence interval does not include the true population mean, while the correct 95% CI spans the whole distribution (as one would expect--with only two independent pieces of information there is little certainty about the true population value). Multiple observations on each rat provides increased precision for estimating the true mean *for that rat*, but does not directly provide increased precision for estimating the population mean in the way that increasing the number of rats does. As the number of samples within each rat increases, the incorrect error bar in Figure 1 will get increasingly narrower, while the correct error bar will remain the same—as it should, because no new information about the population of rats will be obtained by further sampling of these two rats. This idea is also extended to cases where there is more than one experimental group or condition; it is necessary to distinguish between those measurements that are independent samples from the population and which increase precision and decrease uncertainty about the population parameters (which is what the hypotheses tests are testing), and those measurements that only increase the precision of the value for a particular subject.

The second problem that arises is that correlations between observations can lead to calculated *p*-values that are either higher or lower than the true *p*-value. For the above example, "correlation between observations" refers to the degree of similarity of the observations within each rat, relative to the observations between rats. This is called the intraclass correlation (IC) and is expressed as a ratio of variances. We can model the data in Figure 1 as

where *y*_*ij *_are the values of the response, *i *is an index indicating the rat that the observation comes from (*i *= 1 or 2 in this example), and *j *is an index for the observation within each rat (*j *= 1,...,10). The grand mean (the average of the 20 *y *values) is denoted by *μ*, *α*_*i *_is the amount by which the mean of each rat is above or below the grand mean, and *ε*_*ij *_are the residuals, which is the distance of each of the 20 values from the mean of their respective rat. The intraclass correlation can then be calculated as

where  is the variance of the means of the rats about the grand mean, and  is the variance of the residuals (i.e. the unexplained variance). The variability in the data is therefore partitioned into the variability between rats () and the variability within rats (). As can be seen from the above equation, as  gets large, IC approaches zero, and when all the observations within each rat are identical ( = 0), IC approaches one. The IC can thus be interpreted in a similar manner to the Pearson correlation, but restricted to positive values. For the above rat example,  = 83.3 and  = 16.6 giving IC = 0.83, which indicates that the observations within each rat are highly correlated.

A detailed analysis by Scariano and Davenport showed that both the Type I (false positive) and Type II (false negative) error probabilities can be affected by within group correlations [6,7]. When there is a positive within group correlation (the more common situation), the Type I error probability (*α*) will be greater than 0.05, and the greater the correlation the greater the number of false positives. For example, a two independent group comparison with *n *= 10 in each group and with a modest within group correlation of *IC *= 0.30 would give an *α *probability of 0.37; in other words, 37% of the time (and not 5%) the null hypotheses would be (erroneously) rejected. Thus when there is a positive correlation, null hypotheses will be rejected too often, and this is the reason that violating the independence assumption can be more serious than violating the normality or equal variances assumption [8]. The four situations in which pseudoreplication can arise are discussed next and summarised in Table 1.

**Table 1:** Four situations in which pseudoreplication can arise.

Situation	Example	Solutions
Repeated measures	Growth curve	1. Include subject as a random effect
		2. Repeated measures ANOVA
		3. Summary-measure analysis
		
Hierarchical/nested	Multiple brain sections	1. Include random effects
	Multiple coverslips/wells	2. Average over observations
	Litter effects	
		
Correlated in time	Time of day testing occurs	1. Include time as covariate
	Circadian effects	2. Include sample number as a covariate
		
Correlated in space	Multiple incubators	1. Include random effects
	Cage effects	2. Average over observations

### Repeated measurements on the same experimental unit

A common situation is when observations are taken at different times or under different experimental conditions on the same subjects, and this is usually a planned part of the experimental design. Data of this type are typically analysed with a paired-samples *t*-test if there are only two conditions or time points, or a repeated measures (RM) analysis of variance (ANOVA) if there are more than two time points. There are a number of advantages of such designs, including a reduction in the number of animals or participants used, and increased statistical power because subjects act as their own control. The important distinction is that observations from different subjects are independent of each other, but not the observations within each subject. These data are often analysed correctly (in the sense that paired samples *t*-tests are used instead of independent samples *t*-tests), possibly because undergraduate statistics courses for biologists usually cover the difference between "within subjects" and "between subjects" designs.

### Data with a hierarchical structure

A second common design where pseudoreplication can occur is when data are hierarchically organised. Biological data are often sampled at different spatial scales or levels of biological organisation. For example, several brains may be sliced into sections, and a number of regions on a section may be examined histologically (or maybe just the left and right side of the brain), and perhaps only a certain number of cells within each region would be examined. Thus there is a hierarchy, with the whole brain (animal) at the top, sections within a brain, regions within a section, and cells within a region (see reference [9] for a graphical example of hierarchical histological data). If cells are the unit of interest, then typically many cells are examined per brain. Consider an experiment with two experimental conditions (treatment vs. control), with one rat in each condition. The outcome variable is the number of synapses on cells in the CA3 region of the hippocampus, and 100 cells are examined in each rat. This would give 2 rats × 100 cells per rat = 200 data points. The incorrect way to analyse this data is with a *t*-test with an *n *of 200 (similar to the example in Figure 1). This is incorrect because differences due to the treatment are completely confounded with natural animal-to-animal differences between the two rats. The standard deviation (*SD*), in the denominator of the *t*-statistic is meant to represent the variability *between *rats, not within rats. Furthermore, the standard error (*SD*/) is a measure of the uncertainty associated with the means of the *population of rats*, not the populations of cells within rats. The *n *in Equation 1 must therefore represent the number of independent observations, which in this case is the number of rats, not the number of cells(1)

where  is the group mean. Cells within rats will tend to be more similar than cells between rats and therefore are not independent of each other. Including all of the 200 data points in the analysis as if they were independent gives a false estimate of the precision (i.e. the error term is too small) because *t *gets big as  gets big. Two rats will never be exactly the same and therefore it is simply a matter of taking enough measurements on two rats to show that they are statistically different. This point generalises to experiments with more than two groups and more than one factor. If the experiment had used two rats in each experimental condition and 50 cells were observed in each rat, there would still be 200 data points (observations) in total, but the same problem remains, although the treatment effect is not completely confounded with the inter-rat variability.

Another common case of hierarchically structured data is when multiple animals are born in a litter. Animals within a litter are not independent because they share the same parents and the same prenatal and early postnatal environment, and animals are therefore nested within litters [10,11]. Laboratory animals are often highly inbred and genetically identical (or very similar), but epigenetic and developmental factors may play a role, and two rats from the same litter are likely to be more similar than two rats from two different litters, and litter effects have been found on a variety of outcome variables, including life span [2], body weight [12,13], total brain volume (after controlling for body weight [13]), behavioural tests (rotarod [14], possibly prepulse inhibition [15]), and plasma concentrations of various substances (leptin [16], glucose, insulin, triglycerides [17]). It is likely that litter effects are present in many response variables, but few papers mention how these were dealt with in the experimental design stage, or whether the data were examined for the presence of litter effects. If all animals in the control condition are from one litter while all the animals in the treatment condition are from another litter, then the treatment effects will be completely confounded with litter effects, making it difficult to attribute differences between conditions to the effect of the treatment.

Other examples include applying treatments to cages of rats rather than individual rats (e.g. administering a substance in the drinking water), or applying treatments to pregnant females but examining the effect in the offspring. Here, cage and pregnant females are the experimental units, and not the individual animals since the treatments can only be applied to whole cages and pregnant females and not to the individuals animals. This type of experimental design is often referred to as a split-plot design and is characterised by the restrictions on randomisation; it needs to be distinguished from a design where individual rats can be randomised to different conditions. In addition, cells in the same flask or well of a cell-culture experiment are not independent; they will tend to be more similar than cells in different flasks or wells and will be subject to the same uncontrolled effects.

### Observations correlated in space

Observations may be correlated in space because multiple measurements taken at one location will all be affected by the idiosyncratic aspects of that location. For example, 96-well plates often contain small amounts of fluid, and wells near the edges of the plate may evaporate faster than wells in the centre, and thus alter the concentration of substances such as metabolites, secreted hormones, etc. Placing the control samples in the first column of the plate and the treated samples in the second column would therefore not be a good idea. This is also the reason why microarrays have replicate probes for the same gene scattered throughout the array and not placed beside each other, as this accounts for any spatial effects in the quality of the array that may have arisen during manufacturing or handling. Spatial dependence may also arise in incubators for culturing cells. A large cell culture experiment may use two incubators, but differences particular to each incubator may affect the outcome variable. For example, the temperature and humidity levels may be different, or these variables may fluctuate more in one incubator than another, perhaps because one may be used more and thus the door is opened more often as people access their samples. Good experimental design would dictate that the treated samples are not placed in one incubator while the control samples are in the other, as it would be impossible to separate the effect of the treatment from the effect of the incubator.

### Observations correlated in time

Unlike repeated measurements on the same samples, observations that are correlated in time are often not a planned feature of the experimental design, but arise from the sampling protocol, the phenomenon under investigation, or the way in which the experiment is conducted. In addition, observations need not be on the same subject. For example, rats have a circadian rhythm in the stress hormone corticosterone, which peaks at the beginning of the dark (active) phase, and gradually decreases throughout the night [18]. Suppose that plasma corticosterone concentration is the main outcome variable and blood samples from twenty rats need to be taken. If the sampling starts at the beginning of the dark phase (i.e. at the peak concentration) and takes 2 hours to complete, there might be an overall decrease in corticosterone concentration in rats that were sampled at later time points compared to earlier ones. This could confound the results if the first ten rats were the control rats and the next ten were in the treatment group, as it would be difficult to distinguish treatment effects from circadian effects. It would therefore be better to alternate rats from each group when sampling the blood. A circadian effect would not be eliminated, but it could now be taken into account by including time or sample number in the model, which would not be possible if treatment is confounded with time. One example of such a time-dependence between sample number and the main outcome variable is discussed in reference [19].

## Methods

The proportion of papers that had pseudoreplication in a large number of journals was not quantified because the majority of papers do not provide sufficient information for this to be assessed [20]. In addition, the purpose of this paper is not to determine the prevalence of pseudoreplication in the neuroscientific literature but to (1) bring the problem to the attention of the neuroscience community, (2) demonstrate the variety of forms it can take, (3) show how to detect instances of it in publications, and (4) provide alternative analytical methods for dealing with it—and these objectives can be better accomplished with a detailed examination of a few specific papers. It is also in the spirit of Hurlbert's original paper on the topic: "The citing of particular studies is critical to the hoped-for effectiveness of this essay. To forego mention of specific negative examples would be to forego a powerful pedagogic technique" [3].

A single recent issue of *Nature Neuroscience *(August 2008; Volume 11, Number 8) was therefore examined as a "case study". This journal was chosen because it has detailed instructions for reporting the results of statistical analyses (although not always followed), and as a consequence, a greater proportion of manuscripts have sufficient information to assess the analyses. In addition, this is a leading neuroscience journal, and the implication is that if errors of this sort can be found in studies generally considered to be of high quality, then they are also likely to be found elsewhere. This particular issue was chosen simply because of its suitability in illustrating the points being made. It is therefore not necessarily representative of other issues.

The simulated data in Figure 1 was produced with R (version 2.8.0) [21,22].

## Results

Of the nineteen papers published in the August 2008 issue of *Nature Neuroscience*, seventeen papers (89%) used inferential statistics; of these, only three (18%) had sufficient information to assess whether there was pseudoreplication. Of these three, two appeared to have pseudoreplication. Of the fourteen papers that used inferential statistics but did not provide sufficient information, five (36%) were suspected of having pseudoreplication, but it was not possible to determine for certain. A table summarising this information can be found in Additional File 1.

### Manuscripts with pseudoreplication

Fiorillo et al. performed electrophysiological recordings from the brains of two macaque monkeys [23]. We can sympathise with the desire to use as few animals as possible (especially non-human primates), but neurons from the same brain are not independent: they are identical genetically, they have the same developmental history, and they share the same environment, and thus two neurons from the same brain will respond to an experimental stimulus in a similar manner (compared to two neurons from two different brains). Additional technical considerations include neurons receiving inputs from the same structures (perhaps even being innervated by the same neuron), and they can be interconnected either directly via gap junctions or synapses, or indirectly via interneurons. The paper presents data from 42 and 62 neurons (for both monkeys combined; Figure three C and four C in their paper) and inferential statistical tests are performed between two experimental conditions. Unfortunately, the inter-neuron variability is conflated with the inter-animal variability and the analysis must reflect this distinction. It should be noted that this type of analysis is standard in the neurophysiology field, not because it is the optimal approach to address research questions, but because of ethical considerations limiting the number of primates used, and because it is technically easier to record from more neurons in one monkey rather than to record from more monkeys. However, such an experiment with two animals is limited mainly to descriptive statistics such as means and standard deviations.

In another paper, Sato et al. classified rod terminals in the retina as either bipolar or not, and examined whether the proportion of these two terminal types differed between control and pikachurin knockout mice (Figure four E in their paper) using a Chi-square test [24]. The figure caption indicates a total sample size of *n *= 651; however, this is the number of rods and not the number of mice, which is six (*n *= 3 for each genotype). One of the assumptions of the Chi-square test is that observations must be independent; however the factors affecting whether a rod is bipolar or not will tend to affect *all *rods in the same retina in a similar manner. A more appropriate analysis would be to determine the proportion (or percentage) of each rod type for each individual mouse, resulting in six data points that would have values between zero and one. An independent-samples *t*-test could then be used to test whether the mean proportion differed between the knockout and control mice. With a sample size of only six, a *t*-test would only detect large differences between groups and would likely be underpowered. A potential trade-off would be to examine fewer rods in each mouse and examine more mice.

Both manuscripts used hierarchical sampling but did not distinguish between the number of data points and the number of independent observations. These types of errors are not limited to this issue (e.g. see [25,26]) or this journal, but can be found in other top general science journals such as *Cell *[27], *PNAS *[28], and *Science *[29].

### Manuscripts with suspected errors

The following manuscripts possibly had pseudoreplication, but insufficient information was provided to determine for certain. They are nevertheless discussed because they contain other types of analyses where pseudoreplication can arise.

Toni et al. examined how new dentate gyrus neurons integrate and form functional synapses with cells in the hilus and CA3 region of the hippocampus [30]. They examined the size of mossy fibre boutons and reported in the Methods section that 20-21 boutons were analysed per time point in the CA3 region, and between 20-66 were analysed at each time point in the hilus. The results were presented for the CA3 and hilus as *t*_77 _= 10.50, *p *< 0.001 and *t*_156 _= 0.54, *p *= 0.65. There appears to be multiple observations on each mouse, unless the number of mice was greater than 150 (the total number of animals was not stated, but such a large number seems unlikely for this experiment). In addition, the corresponding figure caption (Figure one in their paper) states that the graph displays the means and standard error of the means. However the error bars are so narrow that they are obscured by the data points of the mean values. In both the graph and the analysis there appears to be a misspecification of the structure of the data, and the number of observations (the number of data points) has been confused with the number of independent observations (the number of mice). Other studies by the first author used the same (likely incorrect) analysis [25,31].

Groc et al. examined the effect of corticosterone on AMPA receptor trafficking and synaptic potentiation *in vitro *[32]. The Supplementary Methods state the data were analysed with *t*-tests and ANOVAs (or their non-parametric equivalents) and that 2-6 different sets of hippocampal cultures were used. The figure captions however show very large *n*'s--greater than 1000 in one case. Since degrees of freedom were not provided, it is not known whether values were averaged before analysis, but the large *n*'s suggest that they may not have been.

Pocock and Hobert examined the effects of oxygen levels on axon guidance and neuronal migration in *C. elegans *[33]. The figure captions indicate that *n*'s were typically over 100, and it is also stated that "data were combined from three independent experiments". The data are displayed (Figure one B and one C in their paper) as the percentage of animals with a defect, which suggests that some data reduction has occurred and the values represent the mean percentage over the three independent experiments. However, all of the analyses were conducted with two-sample *z*-tests, which are typically used if the population standard deviations are known, or if the sample size is sufficiently large (the *t *distribution approaches the *z *distribution as *n *→ ∞). Since population standard deviations are not known and need to be estimated from the data, a two-sample *t*-test should have been used if the sample-size was three (i.e. three experiments). Using a *z*-test in such a case leads to an inflated Type I error rate (too many false positives). For example, a test statistic of 1.96 with a *z*-test would give a two-tailed *p*-value just under 0.05 (0.049996), whereas a test statistic of 1.96 with a *t*-test with *n *= 6 (two independent groups of *n *= 3) would give a *p*-value of 0.122, leading to a different conclusion. Therefore either a *z*-test was used where a *t*-test should have been, or a *z *distribution was somewhat approximated by a large *n*, but then the *n *does not reflect the number of independent experimental units. It should be noted that there were very large differences between the groups, and therefore the conclusions are unlikely to change if the data were reanalysed.

Using electrophysiological recordings, Chen et al. examined how the difficulty of a task affected the activity of neurons in the primary visual cortex of two monkeys [34]. The data were analysed with two-sample nonparametric tests (Wilcoxon) and figure captions stated *n*'s for the number of neurons, but not the degrees of freedom, and therefore it is not clear whether each neuron was treated as an independent observation or whether the data were averaged before analysis. The latter is unlikely since there are only two animals.

Serguera et al. examined how dopamine in the olfactory bulb of female mice impairs the perception of social odours contained in male urine [35]. Figure four A in their paper presents data for the amount of time five female mice spent sniffing five different concentrations of male urine (25 observations in total). Each female mouse was exposed to each of the five concentrations of male urine, and the outcome variable was the ratio of time spent sniffing urine versus water. The Methods section only states that the data in the paper were analysed with a two-way ANOVA or *t*-test. Clearly neither of these are appropriate for a one factor experiment with five levels, such as this. Since this is a within-subjects design, where multiple observations are made on each mouse, it is not clear whether the authors used the correct RM ANOVA, or whether they treated the 25 observations as being independent. They report the overall ANOVA analysis as *F*_4 _= 3.4, *p *= 0.02. Given the available information, we can calculate the *p*-values for both a one-way ANOVA and a RM ANOVA (see below for how to calculate these values). A one-way ANOVA with 4 and 20 *df *would give *p *= 0.0282, whereas a RM ANOVA on 4 and 16 *df *would give *p *= 0.0341. Neither analysis corresponds to their reported *p*-value of 0.02, but the one-way ANOVA is closer (perhaps they truncated the value at two decimal places), suggesting that they used the incorrect analysis (the slight discrepancy in calculated *p*-values might also be due to rounding error or differences in the software). Note that discrepancies between *p*-values and their associated test statistics are common, even in top journals such as *Nature *and the *BMJ *[36-39]. The *F *statistic would also be different if the incorrect analysis was used, and so it is not just a matter of different degrees of freedom.

In ambiguous situations such as this, readers have to form some sort of judgement regarding the statistical competence of the authors. Based on other aspects of their data analysis, one may be reluctant to give them the benefit of the doubt. First, an *F*-test has two degrees of freedom associated with it; however, throughout the paper the authors only reported the numerator *df *and not the error *df *(i.e. residual or denominator *df*). This suggests that the authors (and perhaps reviewers and editors) may not be aware that there are two *df*s associated with *F*-tests. Second, in Figure four C in their paper, the authors tested whether the means of *two *groups were significantly different with an ANOVA, and then followed this analysis up with a posthoc test between the same two groups! Not surprisingly, performing the same test twice produced the same result. This is an excellent example of unthinking, rote statistical analysis. The means of two groups can be compared with either a *t*-test or an ANOVA *F*-test, and they will produce identical results; the square of the *t*-statistic is equal to the *F*-statistic, and both have an identical corresponding *p*-value (e.g. for independent groups:  = *F*_(1,*N*-*G*)_, where *N *is the number of independent samples and *G *is the number of groups). Finally, in Figure seven in their paper, the authors present the number of pregnancies carried to term in the female mice under different experimental conditions. They analyse the data with a Chi-square test, but placed asterisks over group number four in the graph. A Chi-square test is an omnibus test, and it provides no information on whether a specific group or condition is different from any other group or condition, yet the authors decorated the figure with asterisks over the group with the lowest number. So did the authors use the correct repeated-measures ANOVA analysis for the data in Figure four A? There is not enough information to know for sure, and this is left for the reader to decide.

## Discussion

In a well-publicised study, Ioannidis concluded that most published research findings in the medical literature are false [40]. One thing he assumed was that at least the statistical analyses were carried out correctly, and inappropriate analyses such as those discussed in the present paper will only increase the number of false conclusions. It is likely that the standard of statistical analysis is much lower in preclinical animal studies, which is due to a variety of reasons, including (1) these studies are less likely to have a statistician associated with them, (2) effects are often large, and the correct conclusions can still be reached no matter how bad the analysis is, (3) they are much less likely to be a part of a meta-analysis [41] and therefore deficiencies in the design, analysis or reporting are not highlighted, (4) the cost of being wrong is minimal since no single animal study will influence the treatment of patients or alter public policy, and (5) experiments are not registered before being conducted, giving greater scope for data dredging and selective reporting. Registering animal experiments has therefore been suggested as a way to improve the quality of animal studies [41-44] or to have "animal subject committees" to "...scrutinise drug trials in animals. The task of such committees would be to assess sample size, randomisation of treatments, blinding of observers, selection of animal subjects, statistical methods... " [45]. These suggestions are not realistic because the goals and therefore methods of preclinical research differ from clinical trials [46]. For example, a typical preclinical grant will contain many small experiments examining many disparate outcomes (e.g. gene expression, behaviour, histopathology, etc.), not one big study with a single primary outcome. Furthermore, later experiments often depend on the results of earlier experiments, and major details may be modified as other studies are published or new methods or techniques become available. Registration and further scrutiny by committees will add a great deal of bureaucracy with only a small improvement in the quality of experiments. Other ways to improve the quality of preclinical studies should be tried first. 

The term pseudoreplication was coined by Hurlbert in 1984 [3], where he argued that current practices in the ecological literature were inadequate, much like Altman did in the medical literature [47]. Since Hurlbert's original publication, pseudoreplication has become less frequent in ecological studies, but has not been eliminated completely [48,49]. A natural question to ask is "how common is pseudoreplication in the neuroscience literature?" Hurlbert found that almost 50% of studies that used inferential statistics had some type of pseudoreplication [3]. Subsequent studies found that the prevalence had decreased to 32% [50] and 12% [48] in the ecological literature. There is no *a priori *reason to think that the neuroscience literature is better than other fields. Indeed ecologists (in general) tend to have greater statistical knowledge than many other biologists (most "statistics for biologists" textbooks are written by those with a background in ecology for example), possibly because they cannot perform highly controlled experiments as laboratory-based scientists can, and so the alternative is to measure potential confounding variables and then take them into account statistically. Given that there has not been much discussion about pseudoreplication in the neuroscience literature, one might speculate that the prevalence is towards the higher end of the scale. There have been some recent papers critical of common statistical practices in the neuroimaging field, where lack of independence is also a central issue [51].

### Reporting guidelines

Medical studies involving human patients have detailed reporting guidelines such as the CONSORT statement [52,53], and there are also guidelines for biological studies such as the *Uniform Requirements for Manuscripts Submitted to Biomedical Journals *by the International Committee of Medical Journal Editors [54]. In 2004 Curran-Everett and Benos provided a set of guidelines for reporting statistics in journals published by the American Physiological Society [55], and in a follow-up paper published three years later they reported that these guidelines had little impact on subsequent practice [56]. Cumming et al. have also made suggestions for describing what error bars in figures should represent [5].

In addition to the above guidelines, further specific information should be provided in order to check whether analyses were carried out correctly. These include:

1. **Report the sample size and number observations for each experiment. **The sample size (*n*) should refer to the number of independent samples and not the number of observations. If there is only one observation per subject then the sample size and the number of observations will be the same. However, if multiple observations are made on each subject, then it is necessary to distinguish the sample size from the observations, as it will allow readers to better understand the design of the study. In addition, the most important reason for the inclusion of these values is that they are necessary to check the results when combined with the information in the next guideline.

2. **Report the value of the test statistic, degrees of freedom, and exact ***p***-value**. These provide the necessary information to check whether the analyses were carried out correctly. They can also allow readers to understand the analysis better if the verbal description was ambiguous. If *p*-values are very small, then *p *< 0.001 would suffice, but not *p *> 0.05, *p *< 0.05, or *p *< 0.01.

**3. Error bars should correspond to the analysis. **Graphical measures of uncertainty such as confidence intervals and standard errors of the mean should be based on the number of independent samples, that is, the graphical representation of the data should correspond to the statistical analysis that was performed on them. If there are two groups of five animals, with multiple observations, the *t*-test should have 10 - 2 = 8 *df *and the error bars should be based on . This is similar to Cumming et al.'s third rule: "Error bars and statistics should only be shown for independently repeated experiments, and never for replicates" [5]. Here they are referring to the fact that one run of the experiment is the experimental unit, and not the number of observations in each experiment (which they refer to as replicates).

Without this information it is not possible for peer-reviewers to adequately assess whether the statistical tests were carried out appropriately, and they must merely assume that the authors have performed the analyses correctly. This is not something that can be safely assumed, and a recent systematic survey identified a number of problems with the reporting, experimental design, and statistical analysis of studies using laboratory animals [20]. These guidelines need to be made requirements for publication and therefore the initial responsibility lies with journal editors. The *Nature *series of journals have already improved their reporting requirements after a study showed numerous errors in one of their journals [36], but there is still room for further improvement. The *European Journal of Neuroscience *has also recently issued guidelines which are similar to those suggested above [57], and these recommendations could be easily adopted by other journals. Until journal requirements change, reviewers must insist on this information being provided. Including all of the above information might make the text difficult to read, especially if a number of results are presented in succession. All of this information therefore need not be presented in the main text, and the full results can be reported in the online supplementary material (which is available for most, if not all journals), and the main text could include only *p*-values and the sample size for example.

### Remedies for pseudoreplication

Pseudoreplication does not necessarily imply that the studies are flawed, and a reanalysis of the data may be all that is required. It may however become apparent that the sample size is too small to make any meaningful inferences about the parameters of interest. Pseudoreplication can be dealt with prior to analysis, for example by using only one mouse per litter for a particular experiment, thus eliminating any litter effects. Statistical methods for dealing with pseudoreplication are available and four such methods are discussed below and summarised in Table 1. Other options for ecological studies are discussed by Millar and Anderson [58].

#### Averaging dependent observations

In the opening example with ten rats undergoing rotarod testing on three consecutive days, the results from the three days can be averaged so that each rat contributes only one value to the analysis. This is particularly useful when there is no expected trend over the days of testing, or if there is, it is not relevant to the research question; for example, if three trials were simply used to get a better estimate of the rats' motor functioning. Similarly, in a hierarchical sampling design, one could average values from multiple neurons in a rat to obtain one value per rat that will then be carried forward for statistical analysis. Averaging has the advantage of simplicity, and common statistical tests can be applied (e.g. *t*-test, ANOVA). A drawback is that information is lost when averaging; for example, there may be a different number of observations for each rat, and the observations for some rats might be more variable than for others. Therefore some estimates of the mean response for each rat are more precise than others, but this information is not used in the analysis and each mean value is treated equally, rather than being weighted according to its precision. It should also be noted that averaging can lead to bias when the number of observations is correlated with the outcome variable, however this is more of a concern for observational studies and longitudinal clinical studies. This is not a major concern for laboratory-based experimental studies, where the number of observations is under control of the experimenter, and any missing values typically occur at random.

#### Summary-measure analysis

Another alternative to using the mean of a number of dependent observations is to use some other relevant value which captures a feature of interest, such as the slope, intercept, or area under the curve [59]. This is referred to as summary-measure analysis or derived-variable analysis. Using the same rat example, suppose the researcher was interested in whether there was a change in rotarod performance over the three days, such as a practice effect. For each rat, a regression analysis could be carried out using day as the explanatory variable (*x*) and time spent on the rotarod as the response variable (*y*). The slope of the regression line would then be used for further analysis, perhaps to compare whether one group improved faster than another, and in the absence of a practice effect, the values of the slopes should be centred around zero. As above, it has the advantage of reducing many correlated observations to fewer independent observations, it is conceptually straightforward, and it allows for the use of standard tests. The drawbacks are also the same, namely, information is lost. In addition, since there were only three time points, the estimates of the slopes would have low precision (one slip by a rat and the slope for that rat can change dramatically). This analysis is not the recommended one and is only given for completeness. An alternative analysis that could be used is a repeated-measures ANOVA, where day would be the within-subjects factor and condition (treatment vs. control) would be the between subjects factor.

There is also an important point to be made when deciding whether to average over observations or to use slopes as a summary measure (or a mixed model), and it is based on whether the research question is (1) do subjects with high values of *x *also have high values of *y*, or (2) within each subject, are high values of *x *associated with high values of *y*. These are different questions and are discussed in a series of papers by Bland and colleagues [59-62]. In the first case, average all the *x *and *y *observations on each subject, and then use these for analyses. In the second case, analysis of the slopes or mixed models can be used.

#### Separate analyses

Another option is to conduct separate analyses on each of the three days, using an independent samples *t*-test at each day. This means that more than one statistical test is performed, raising the issue of whether corrections for multiple tests should be made. Furthermore, there is no integrated final result, only a collection of disjointed *p*-values that may be difficult to interpret. What would the interpretation be if there was no significant difference between groups on the first or third day of testing, but there was a significant difference on the second day? Should we conclude that there is a treatment effect because there was at least one significant *p*-value, or should we take a majority vote: two non-significant versus one significant *p*-value means that the treatment really did not have an effect (and the unusual significance of only the middle value considered a false positive)? It is complicated by the significant result being the middle day and not the first or last day (we might have explained the results in terms of practice effects). Separate analyses are therefore not very useful, but they are often performed to test whether there are significant effects at each time point after a test for an overall main effect. This goes by the name of posthoc testing, which is routinely performed and mostly unnecessary [63]. It is not uncommon to have a significant main effect of treatment and then have none of the posthoc tests significant. This is due to the reduced power of the posthoc tests.

#### Mixed models

As noted above, a repeated measures ANOVA is a common analyses for the rotarod example if there was interest in testing for differences across the three days; however, this method has been superseded by more recent methods with superior properties that are called random (or mixed) effects models, hierarchical models, multilevel models, or nested models (different disciplines use different names for the same method) [64]. These are similar to the averaging and summary-measure methods discussed above, but instead of performing the analysis in two steps (e.g. calculating the slope for each rat, and then performing the analysis on the slopes) the analysis is performed in one step. One important advantage is that information on the precision of the estimates is retained and used in the analysis

A key feature of these models is the distinction between fixed and random effects. Fixed effects are the familiar explanatory variables such as treatment, sex, condition, and dose, and are usually something that the experimenter is interested in testing directly. Fixed effects affect the mean of the outcome variable; for example, the effect of a treatment is to increase the value of the outcome variable compared to a control condition by a certain number of units. Random effects are less familiar and are usually something that the experimenter is not interested in directly (litter effects, cage effects, differences between incubators, differences between individual rats, etc.) but must be taken into account. A variable can be treated as being either fixed or random, but usually one is more appropriate, and the interpretation of the results is different. For example, if a researcher was interested in testing whether there are differences between cages on some outcome variable, twenty rats could be randomly assigned to four cages (5 rats per cage), labelled A-D. In this example there is no other experimental variable, only the cage that the rat is in. Treating cage as a fixed effect would lead to a one-way ANOVA with four levels. If significant differences are found between cages, then conclusions can only be made about these four cages, and not about other unobserved cages. If rats in cage C had particularly high values, there is no reason why in a subsequent experiment rats in a cage also labelled C would also have high values, rather than rats in cage B for example. There is nothing about the letter C on the front of the cage that affects the mean value of rats in that cage, or that can be used to predict the value of rats in other cages also labelled C; thus the cage labels are said to be uninformative. Contrast this with a true fixed effect such as dose of a drug; if the 50 mg/kg group had higher values than the 0 mg/kg control group, then one would also expect that the 50 mg/kg group would have the higher values in a subsequent experiment (rather than the 0 mg/kg group). If instead cage is treated as a random effect (the more appropriate analysis), then these four cages are treated as random samples from a population of cages, and inferences can be made about the effect of cages in general. A good discussion of the difference between fixed and random effects can be found in references [65] and [66].

One important drawback of the repeated measures ANOVA is that the assumptions of compound symmetry and sphericity are rarely met. These terms refer to the correlation structure of the data. Returning to the rat rotarod example, the correlation of the outcome variable at each combination of time points can be calculated (day one vs. day two, day one vs. day three, and day two vs. day three). If these three correlations are all similar, and in addition the variances at each day are similar (homogeneity assumption), then the data are said to be compound symmetrical. If differences rather than correlations between each combination of time points are calculated, then the data are said to be spherical if these difference scores all have the same variance (see reference [67] for a discussion). These assumptions are usually not met because observations closer in time tend to be more highly correlated than observations further apart. The traditional solution has been to adjust the degrees of freedom of the *F*-statistic so that the actual *α*-level is closer to the nominal *α*-level (few papers actually mention using any correction, such as the Greenhouse-Geisser or Huynh-Feldt). Modern statistical methods can model different types of variance-covariance relationships directly, making *ad hoc *adjustments to degrees of freedom unnecessary. Kristensen and Hansen provide an excellent introduction and a comparison of different analyses on rats [68] (and also a nice graph illustrating the correlation structure of the data) and Gueorguieva and Krystal discuss the advantages of mixed models over repeated measures ANOVA [64]; introductory books include Zuur et al. (with biological examples; [69]), Crawley [65,70] and Faraway [71], and a more comprehensive treatment can be found in Pinheiro and Bates [72].

Mixed models and their extensions (generalised and nonlinear mixed models) are the preferred methods for analysing the type of data discussed in this paper and are already being used to model litter effects [13] and hierarchically structured data [9]. The above methods do not exhaust the possibilities for dealing with data of this type, but highlight some of the more common methods and their advantages and disadvantages.

### How to check reported values

When the necessary information is provided, it is easy to check whether pseudoreplication has been handled correctly, for this one needs to know the degrees of freedom associated with common statistical tests, and these are provided in Table 2 for reference. The hypothetical rotarod example in the introduction can now be easily checked. The authors stated that there were ten rats and that they used an independent samples *t*-test; Table 2 indicates that the correct number of *df *= 5 + 5 - 2 = 8, and not the 28 that the authors reported. Even if degrees of freedom are not reported, it is still possible to determine whether pseudoreplication was handled correctly using standard software such as OpenOffice Calc or MicroSoft Excel. Both of these programmes have a TDIST function which can calculate a *p*-value given a *t*-statistic, degrees of freedom, and whether the test is one or two-sided. If the *df*s were not reported for the rotarod example, we could check the results with =TDIST(2.1; 8; 2), where 2.1 is the reported *t*-statistic (note that only the absolute value is need; the negative sign can be omitted), 8 is the correct number of degrees of freedom, and 2 indicates that it is a two-tailed test. The result is *p *= 0.07, which does not correspond to the reported value of *p *= 0.045. Substituting 28 *df *for 8 in the above command does give *p *= 0.045, and allows us to conclude that the reported analysis is incorrect. The same procedure can be carried out in R using the command pt(q = 2.1, df = 8, lower.tail = FALSE)*2 (again, only the absolute value of the *t*-statistic is needed).

**Table 2:** Degrees of freedom associated with common statistical tests.

Test	Degrees of Freedom
**T-test**	
Independent	*n*_1 _+ *n*_2 _- 2
Paired	*n *- 1
	
**One-way ANOVA**	*G *- 1 and *n *- *G*
	
**Two-way Anova**	
Main effect of A	*G*_*A *-1_
Main effect of B	*G*_*B *-1_
A × B interaction	(*G*_*A *-1_)(*G*_*B *-1_)
Error	*n *- *G*_*A*_*G*_*B*_
	
**One-way RM-Anova**	
Between subjects	*G *- 1
Error	(*n *-1)(*G *-1)
	
**Two-way Mixed^**† **^ANOVA**	
*Between subjects*	*n*(*G *- 1)
Groups	*G*-1
Error	*G*(*n*-1)
*Within subjects*	*N - nG*
Obs	*Obs - *1
Group × Obs Interaction	(*G *- 1)(*Obs *- 1)
Error	*N - nG - G *(*Obs *- 1)
	
**Linear Regression**	1 and *n *- 2
	
**Chi-square**	(*R *- 1)(*C *-1)

*n*_1 _= sample size of group one; *n*_2 _= sample size of group two; *n *= total sample size; *N *= total number of observations (equal to *n *× *Obs*); *G *= number of groups; *G*_*A *_= number of groups for Factor A; *G*_*B *_= number of groups for Factor B; *Obs *= number of repeated observations on the same subject; *R *= number of rows; *C *= number or columns. † "Mixed" refers to the presence of both between and within subjects effects, and should not be confused with mixed effects models described in the text.

A similar procedure can be carried out for ANOVA *F*-tests using the =FDIST(test statistic; df1; df2) function in OpenOffice/Excel and pf(q =, df1 =, df2 =, lower.tail = FALSE) in R/S-Plus. This analysis may not be of much use if the authors only report whether the *p*-value was greater or less than 0.05, but it does provide a method for checking partially reported results.

## Conclusions

The problem of pseudoreplication has been recognised for many years in ecology and related areas [3], as well as in the medical literature, where Altman writes "In some conditions it is possible to take several measurements on the same patient, but the focus of interest usually remains the patient. Failure to recognise this fact results in multiple counting of individual patients and can lead to seriously distorted results. Analysis ignoring the multiplicity violates the widespread assumption of statistical analyses that the separate data values should be independent. Also, the sample size is inflated, sometimes dramatically so, which may lead to spurious statistical significance" [73]. The neuroscience community has not recognised the importance of dealing with this type of data appropriately, although a recent paper has highlighted the negative effect this can have on translational research [2].

Statistical competence will not happen overnight, but stricter reporting requirements will make it easier to detect pseudoreplication, and this requires the sample size (*n*), degrees of freedom, the test statistic, and precise *p*-values to be reported, allowing many of these errors to be detected at a glance. This information also allows for a more detailed analysis if required, either by editors, reviewers, or other readers.

Continuing with the present situation suggests that statistical analysis is not really important, it's just something scientists go through to obtain *p*-values that can be tacked on to the end of sentences, or to calculate the number of asterisks that can be used to decorate a graph. Many medical advances are based on preclinical animal research, it is therefore important that preclinical studies are conducted, analysed, and reported correctly.

## Author information

SEL's academic (BA, BSc, PhD) and research background are primarily in experimental neuroscience. SEL also holds a masters in Computational Biology and currently works as a Senior Research Statistician in the pharmaceutical industry.

## Response to Lazic

by Christopher D. Fiorillo

Email: fiorillo@kaist.ac.kr

Address: Department of Bio and Brain Engineering, Korea Advanced Institute of Science and Technology, Daejeon, Korea

Lazic makes many useful points about the misuse of statistics. However, his article on pseudoreplication does not demonstrate a clear understanding of the goals and issues at stake in primate neurophysiology, nor does it clarify the importance of independence in statistical tests.

Lazic's criticisms of my paper [23] generalize to virtually all papers within the field of primate neurophysiology, in which the standard is to record from many neurons, but in only two monkeys. That the field has been able to make substantial progress is largely dependent on there being relatively little variation from one brain to another in most of the phenomena that have been examined. (Although there is substantial variability in responses across "trials" and across neurons). The interest in virtually all and papers is inacross responses within and across neurons, not in comparisons across animals. Of course there could always be differences between individual animals, and it is important not to conflate variation across neurons with variation across animals. My manuscript may indeed have given the appearance of conflating the two. The data was in fact presented separately for the two animals in the original manuscript, but during the review process it was combined for the sake of simplicity, as is common within the field. The standard is that statistical significance should be demonstrated separately within each monkey. I did this for each monkey, although I only stated this in the main text with respect to figure four c, but not with respect to figure three c.

The statistic comparison that the author has questioned (in figures three c and four c) was designed to test whether there is a difference in neuronal firing rates (across the recorded population of neurons) between responses to juice reward depending on when juice was delivered following a conditioned stimulus. If one knows the subject matter and reads my paper for more that just statistical methods, then one has a strong prior expectation that the statistical comparisons made in figures three c and four c will be highly significant, and thus the statistical significance is not of much interest. The interesting point of the figures, as described in the main text, is that although significant, the differences are surprisingly small relative to the very large difference seen in comparing either of these responses to unpredicted reward. The statistical significance that was quantified in these figures was thus superfluous and could have been omitted entirely. There were no statistical tests performed on the important effect because it was so large as to be obvious (see mean ± sem firing rates in figures three b and four b). To paraphrase a colleague who is an excellent scientist but a reluctant statistician, it passed the "bloody ... obvious test."

Lazic also makes the point that "neurons of the same brain are not independent." If this statement does not signify confusion on the part of its author, it may nonetheless confuse readers. There is an important difference between physical or causal independence on the one hand, and logical or statistical independence on the other. Neurons in the same brain may be physically or causally related. However, that fact in itself does not necessarily require that the neurons are or are not statistically or logically independent. Statistical independence is all that matters with respect to statistical tests, and statistical dependencies could be present regardless of whether the neurons are in the same brain or different brains.

A second critical error that is often made is to confuse the functioning of a physical system with our knowledge of that system. Statistics and hypotheses are derived from the latter. "Statistical independence" means that we, the people performing the statistical test, do not have knowledge of how two pieces of data (such as the firing rates of two neurons) are related. We believe that neurons within a defined population, clustered together in the same region of the brain, are likely to have direct or indirect physical interactions with one another, and likewise, to display some sort of correlations in their firing rates. But at the outset of a typical study, we do not know what these correlations are, and thus it is rational for us to treat the data from each neuron as independent. By contrast, if we already knew neurons within clusters of known dimensions to be tightly coupled to one another through gap junctions, then we would probably try to avoid recording from neurons within the same cluster, and when we did obtain data from neurons in the same cluster, we might average it together before doing an analysis of responses across clusters. The data from my neurons was "statistically independent" because, at the time that I did the statistical test, I was ignorant of any relevant relationship between the firing rates of discrete neurons. Given a different state of knowledge, statistical independence may not apply and another type of statistical test may be more appropriate.

I strongly recommend *Probability Theory, the Logic of Science*, in which E.T. Jaynes discusses these issues and other "pathologies of orthodox statistics other "path " [74]. It will be clear to anyone reading his text that there are fundamental disputes within the field of probability and statistics that have yet to be fully resolved. Given that information and probabilities are inherently linked, this topic is of particular importance in studying information processing within the nervous system [75].

### Acknowledgements

C.D.F. was supported by a "World Class University" Grant from the Korea Science and Engineering Foundation (R32-2008-000-10218-0).

## Signed response

By Takahisa Furukawa, M.D and Ph.D.

E-mail: furukawa@obi.or.jp

Address: Department of Developmental Biology, Osaka Bioscience Institute, 6-2-4 Furuedai, Suita, Osaka, 565-0874, Japan

Many life science studies employ histological analyses, frequently immunostaining of tissue sections, and incorporate this image data into papers. Although it used to be that only the most representative images were displayed in paper figures, quantitative analysis along with statistical analysis of data are often required to obtain an analytical conclusion on immunostaining image data for publication. The author, Lazic, mentioned in his paper that the reason he picked Nature Neuroscience in particular for his case study is that this journal has detailed instructions for statistical analyses. As Lazic mentioned, authors basically examine their results to test whether or not their data is statistically significant, and present their data with statistical analysis results in their papers. This means that this journal requires high quality data and expects researchers conduct their studies according to these requirements. In Lazic's paper, the author picked multiple examples, In including our statistical analysis of our electron microscopy (EM), and indicated that the statistical analysis method used in our paper was inappropriate [24]. Lately, electron microscopic (EM) analysis of the ultra-microstructure of nervous tissues and neurons is employed as one type of histological data in many papers. However, EM analysis is in general very time consuming, technically difficult, expensive, and limited by the availability of EM devices. Thus, it should be noted that acquiring a large enough number of samples for EM analysis is not easy. I suppose that, for these reasons, many EM studies are not accompanied by statistical analysis, or do not provide sufficient information about their statistical analysis. However, even under these conditions, we examined more than two hundred ribbon synapses for both the wild-type retina and the pikachurin null retina from three mice. We counted as many synaptic terminals as possible to increase the reliability of our analysis. First, I would like to emphasize that these large numbers in our EM analysis reflect our sincere attitude to conduct quality scientific research. Second, we used the Chi-square test to analyze our EM data and determine using EM images if the lack of bipolar terminus invagination in photoreceptor ribbon synapses of the pikachurin null retina is statistically significant compared to those of the control retina. Lazic mentioned that the t-test should have been used instead of the Chi-square test for this analysis, because the Chi-square test is used for observations that are independent. We thought that some aspects of our EM data might correspond to independent observations. In the wild-type retina, basically all photoreceptor ribbon synapses are invaginated by the bipolar terminus. However, in the EM analysis, which uses ultra thin sections for observation, a significant number of sections seem to lack the invagination of bipolar dendritic tips even in the wild-type retina. We observe the very ends of the dendritic tips and thus the appearance of the ends depends on section angles that are uncontrollable in EM analysis. In this case, we thought that the appearance of invaginated bipolar cell dendritic tips on EM sections could be considered random and independent. Thus, we used the Chi-square test to analyze our results. Since we were not completely sure that our EM section analysis really corresponded to random events after reading the Lazic paper, as Lazic suggested, we re-calculated the percentage of invaginated rod terminals by bipolar dendritic tips for each individual mouse. We re-analyzed the data by using the independent-samples t-test and obtained a similar result to that of the Chi-square test. In this statistical analysis, we found that 3% of rod terminals contain invaginated bipolar terminals in the pikachurin null retina, whereas 56% of rod terminals contain invaginated bipolar terminals in the wild-type retina (t-test, P < 0.01).

In order to confirm this EM observation, we also performed 3D electron tomography analysis and described the result in the paper. Furthermore, the results from other experiments including electroretinogram and optokinetic responses also support our conclusion that photoreceptor synaptic terminal formation is impaired in the pikachurin null retina. Ultimately, the Lazic paper points out some very important issues in conducting appropriate statistical analysis for biological studies. We understand that we have to be very careful in choosing a suitable statistical method for analyzing our data in the future, however, the Lazic paper does not affect the conclusions in our paper.

## Supplementary Material

Additional file 1 **Classification of manuscripts**. Summary of the papers in the August 2008 issue of *Nature Neuroscience*. Click here for file
